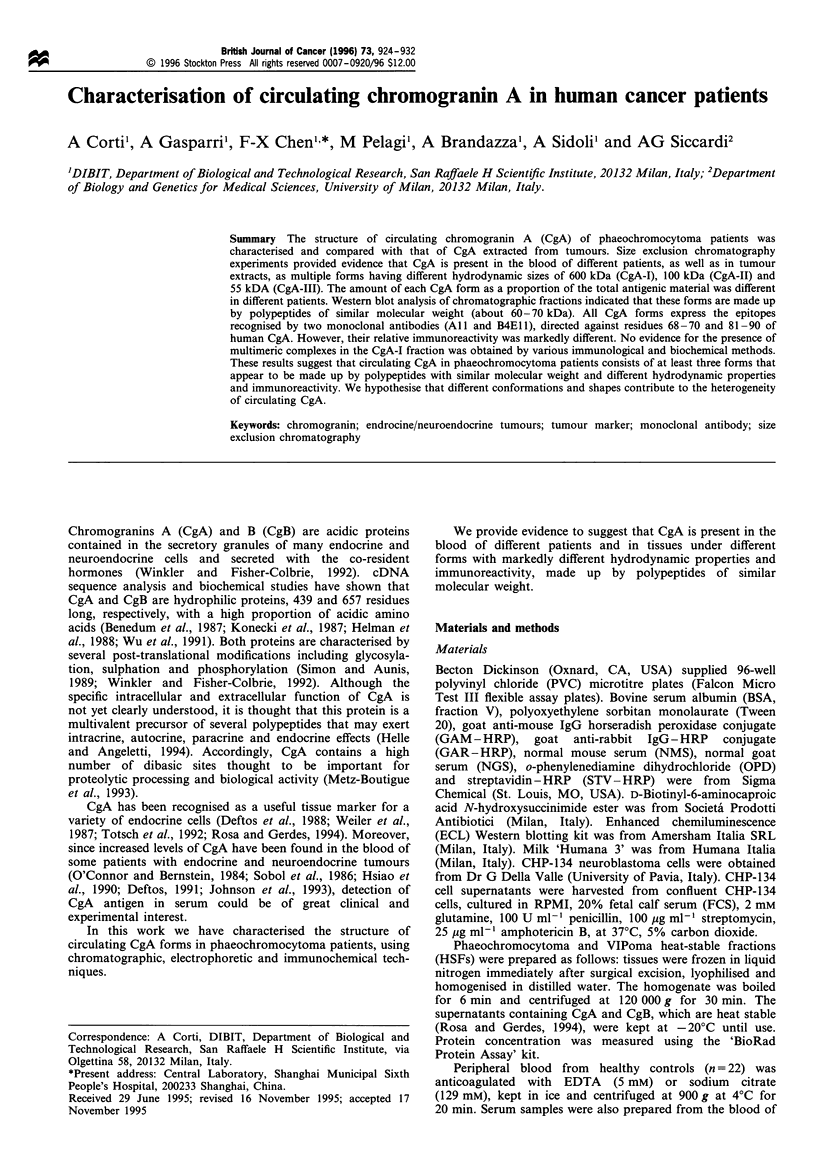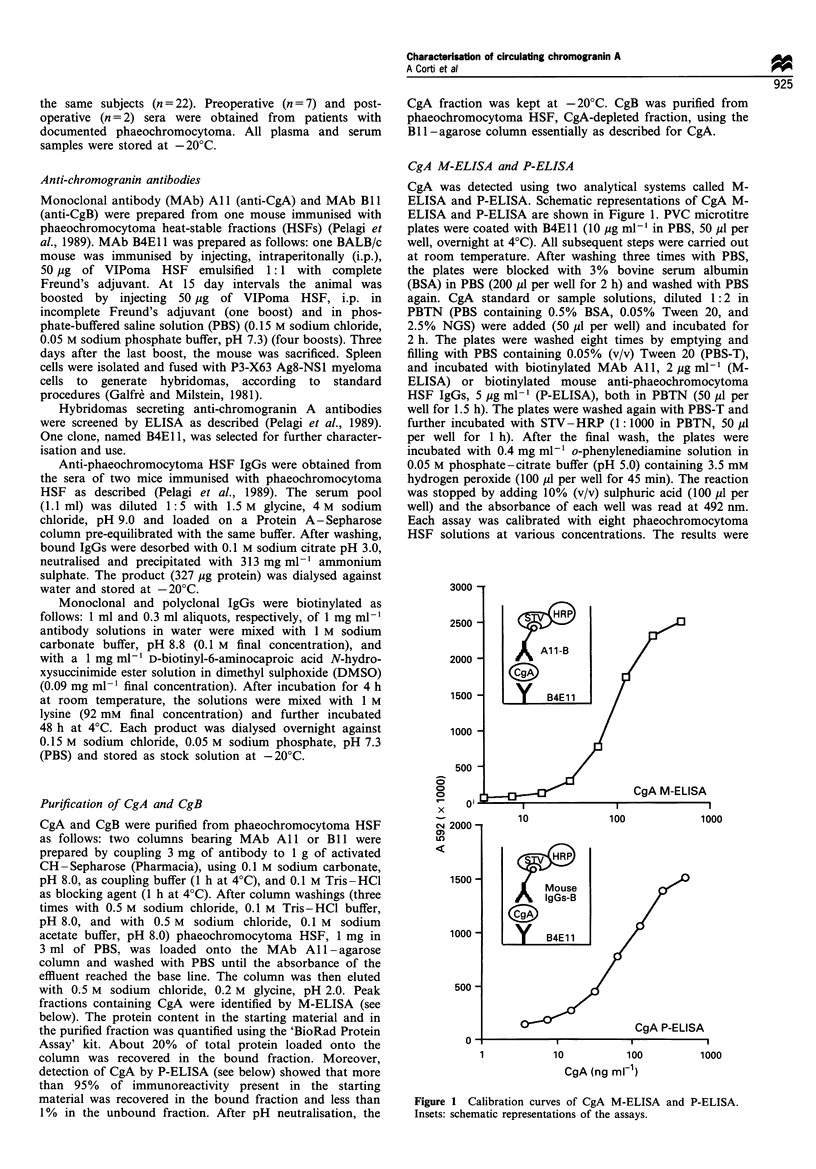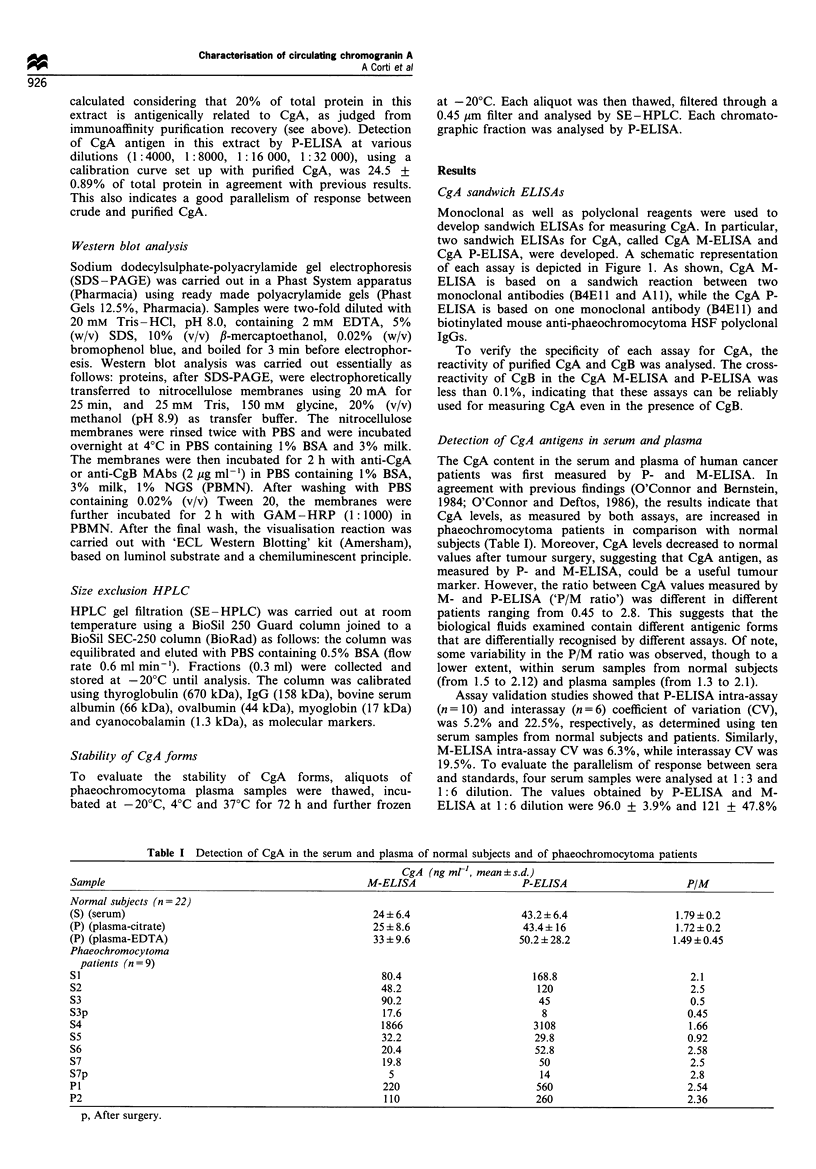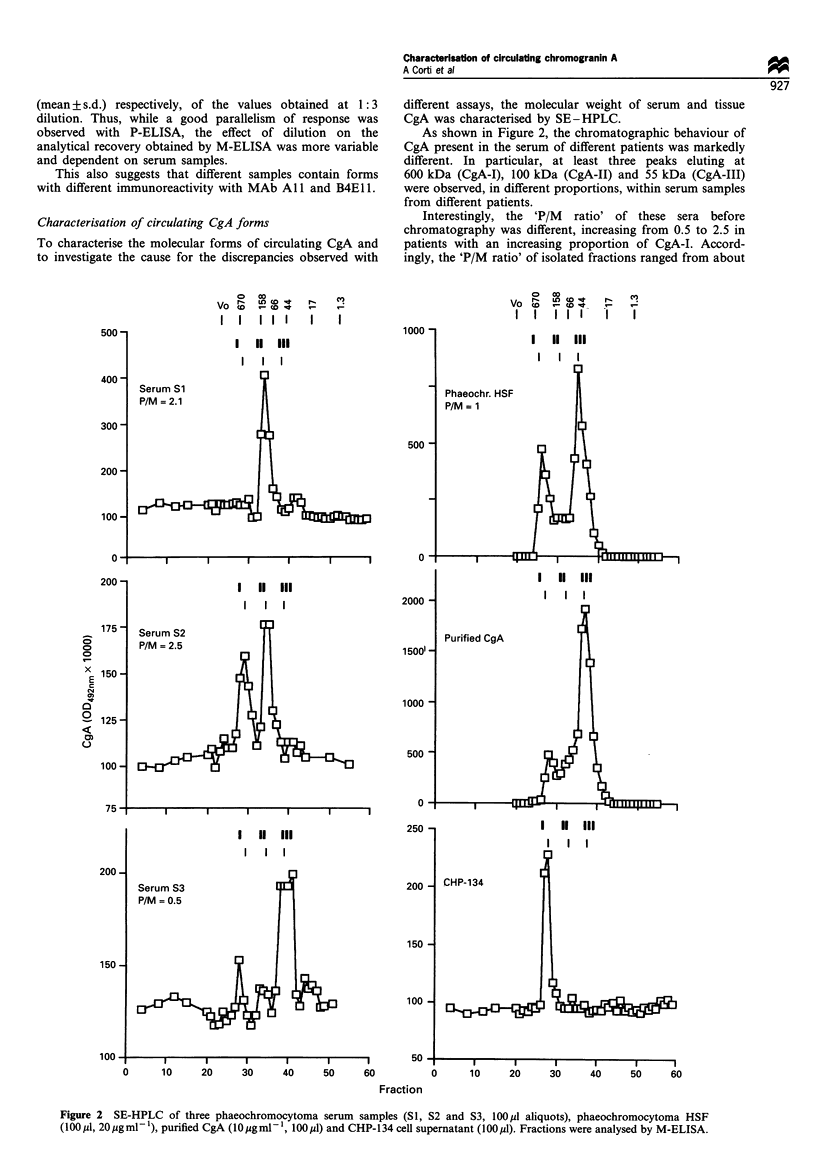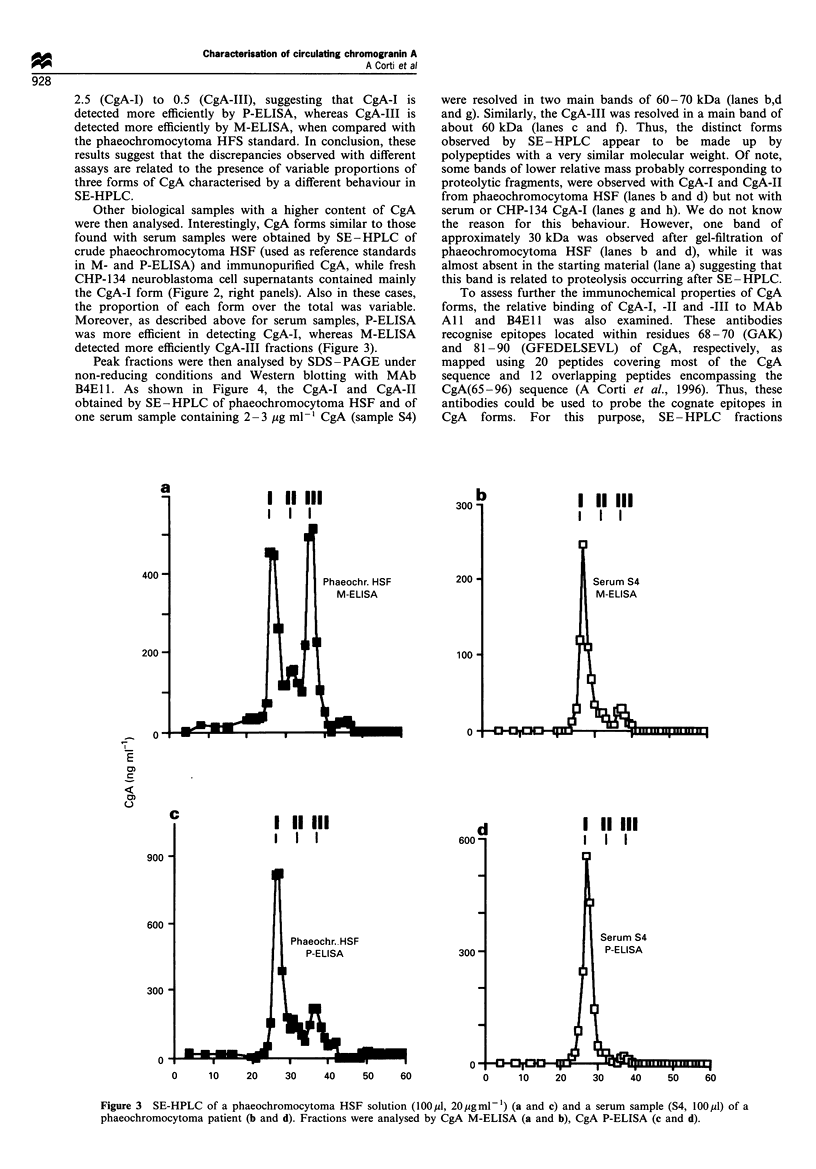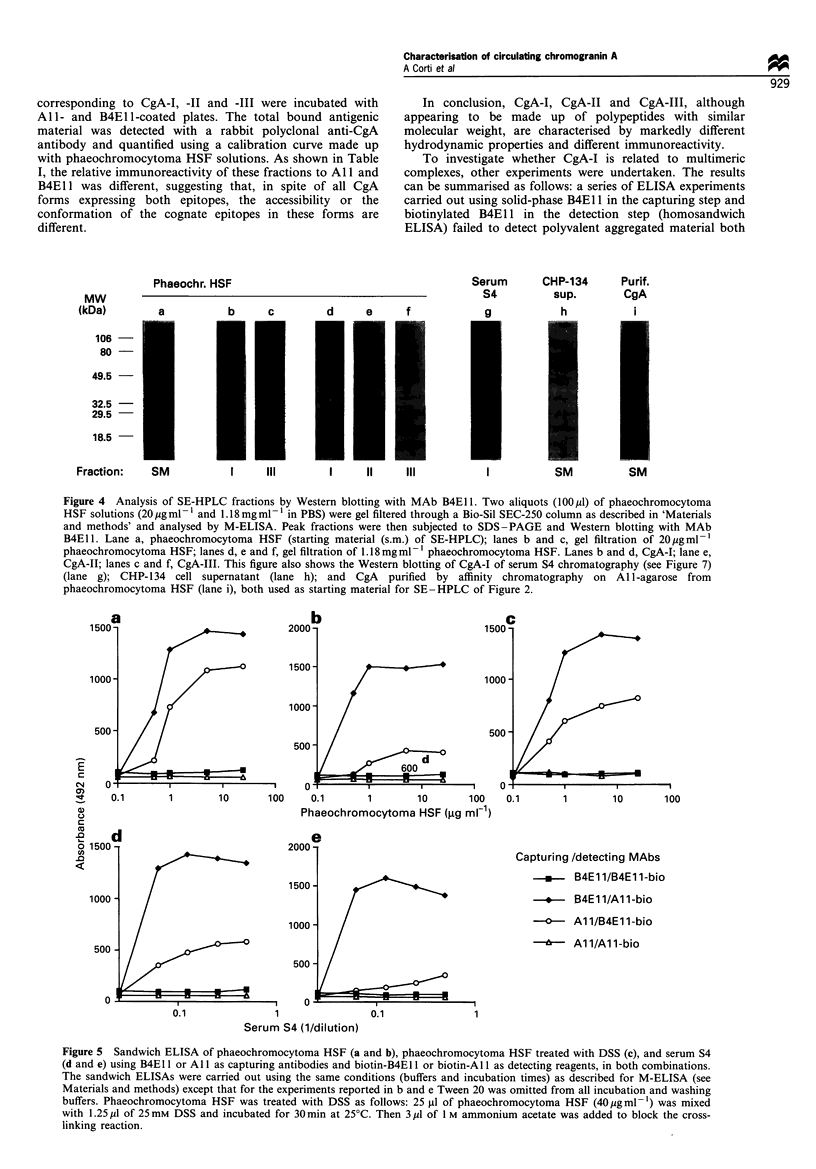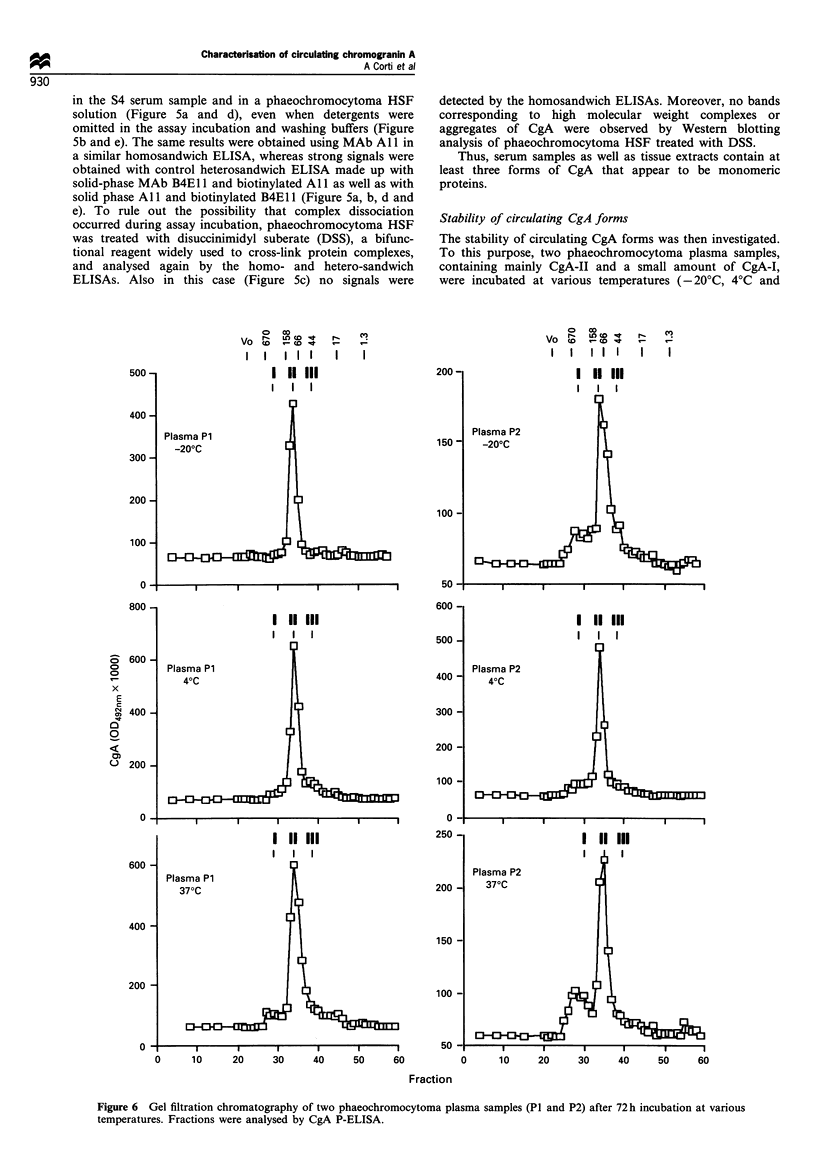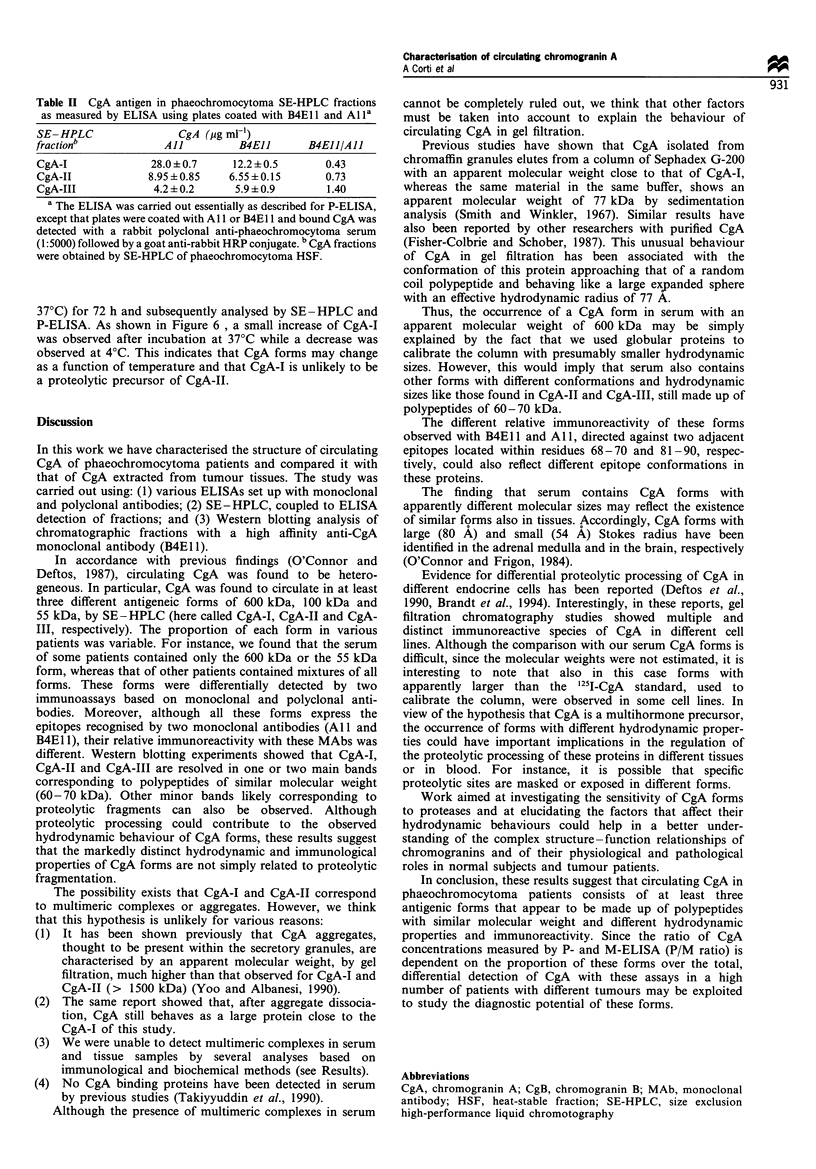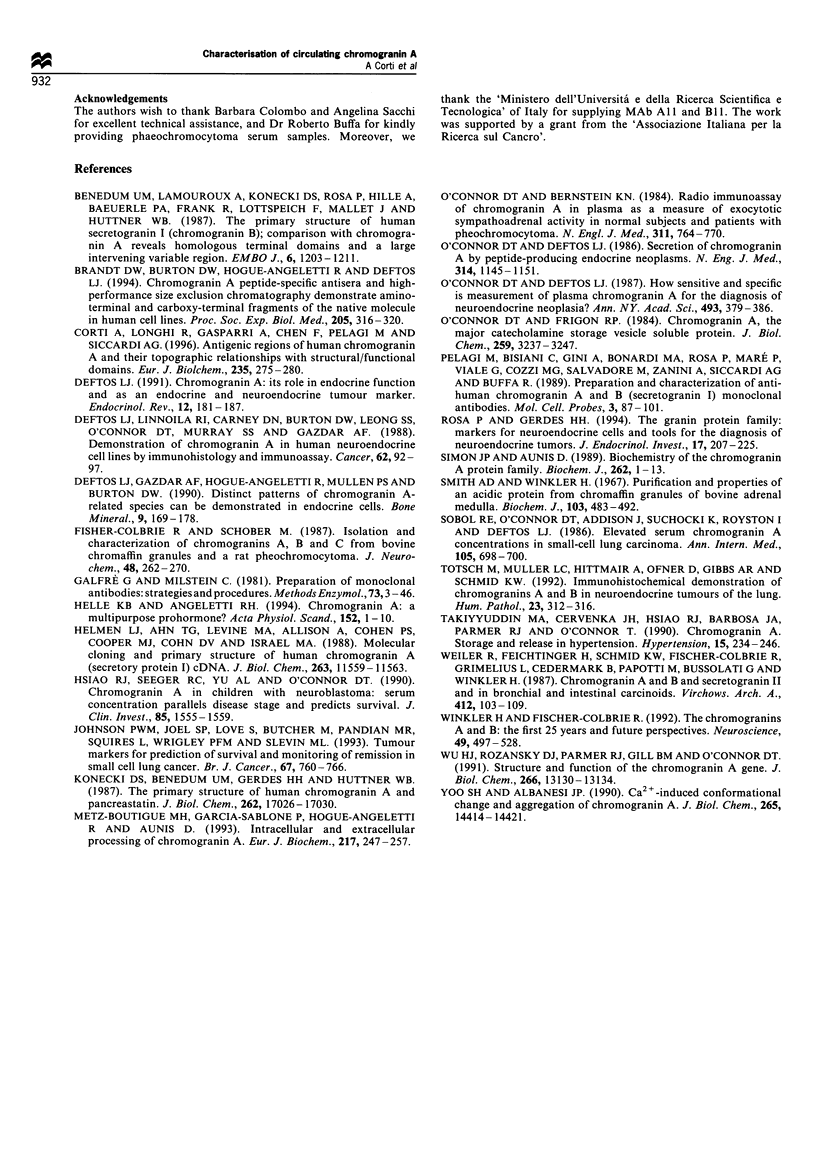# Characterisation of circulating chromogranin A in human cancer patients.

**DOI:** 10.1038/bjc.1996.183

**Published:** 1996-04

**Authors:** A. Corti, A. Gasparri, F. X. Chen, M. Pelagi, A. Brandazza, A. Sidoli, A. G. Siccardi

**Affiliations:** DIBIT, Department of Biological and Technological Research, San Raffaele H Scientific Institute, Milan, Italy.

## Abstract

**Images:**


British Journal of Cancer (1996) 73, 924-932
© 1996 Stockton Press All rights reserved 0007-0920/96 $12.00

### Characterisation of circulating chromogranin A in human cancer patients

A Corti[1], A Gasparri[1], F-X Chen[1,*], M Pelagi[1], A Brandazza[1], A Sidoli[1] and AG Siccardi[2]

[1]*DIBIT, Department of Biological and Technological Research, San Raffaele H Scientific Institute, 20132 Milan, Italy;* [2]*Department of Biology and Genetics for Medical Sciences, University of Milan, 20132 Milan, Italy.*

**Summary** The structure of circulating chromogranin A (CgA) of phaeochromocytoma patients was characterised and compared with that of CgA extracted from tumours. Size exclusion chromatography experiments provided evidence that CgA is present in the blood of different patients, as well as in tumour extracts, as multiple forms having different hydrodynamic sizes of 600 kDa (CgA-I), 100 kDa (CgA-II) and 55 kDA (CgA-III). The amount of each CgA form as a proportion of the total antigenic material was different in different patients. Western blot analysis of chromatographic fractions indicated that these forms are made up by polypeptides of similar molecular weight (about 60-70 kDa). All CgA forms express the epitopes recognised by two monoclonal antibodies (A11 and B4E11), directed against residues 68-70 and 81-90 of human CgA. However, their relative immunoreactivity was markedly different. No evidence for the presence of multimeric complexes in the CgA-I fraction was obtained by various immunological and biochemical methods. These results suggest that circulating CgA in phaeochromocytoma patients consists of at least three forms that appear to be made up by polypeptides with similar molecular weight and different hydrodynamic properties and immunoreactivity. We hypothesise that different conformations and shapes contribute to the heterogeneity of circulating CgA.

**Keywords:** chromogranin; endrocine/neuroendocrine tumours; tumour marker; monoclonal antibody; size exclusion chromatography

Chromogranins A (CgA) and B (CgB) are acidic proteins contained in the secretory granules of many endocrine and neuroendocrine cells and secreted with the co-resident hormones (Winkler and Fisher-Colbrie, 1992). cDNA sequence analysis and biochemical studies have shown that CgA and CgB are hydrophilic proteins, 439 and 657 residues long, respectively, with a high proportion of acidic amino acids (Benedum *et al.*, 1987; Konecki *et al.*, 1987; Helman *et al.*, 1988; Wu *et al.*, 1991). Both proteins are characterised by several post-translational modifications including glycosylation, sulphation and phosphorylation (Simon and Aunis, 1989; Winkler and Fisher-Colbrie, 1992). Although the specific intracellular and extracellular function of CgA is not yet clearly understood, it is thought that this protein is a multivalent precursor of several polypeptides that may exert intracrine, autocrine, paracrine and endocrine effects (Helle and Angeletti, 1994). Accordingly, CgA contains a high number of dibasic sites thought to be important for proteolytic processing and biological activity (Metz-Boutigue *et al.*, 1993).

CgA has been recognised as a useful tissue marker for a variety of endocrine cells (Deftos *et al.*, 1988; Weiler *et al.*, 1987; Totsch *et al.*, 1992; Rosa and Gerdes, 1994). Moreover, since increased levels of CgA have been found in the blood of some patients with endocrine and neuroendocrine tumours (O'Connor and Bernstein, 1984; Sobol *et al.*, 1986; Hsiao *et al.*, 1990; Deftos, 1991; Johnson *et al.*, 1993), detection of CgA antigen in serum could be of great clinical and experimental interest.

In this work we have characterised the structure of circulating CgA forms in phaeochromocytoma patients, using chromatographic, electrophoretic and immunochemical techniques.

Correspondence: A Corti, DIBIT, Department of Biological and Technological Research, San Raffaele H Scientific Institute, via Olgettina 58, 20132 Milan, Italy.

*Present address: Central Laboratory, Shanghai Municipal Sixth People's Hospital, 200233 Shanghai, China.

Received 29 June 1995; revised 16 November 1995; accepted 17 November 1995

We provide evidence to suggest that CgA is present in the blood of different patients and in tissues under different forms with markedly different hydrodynamic properties and immunoreactivity, made up by polypeptides of similar molecular weight.

### Materials and methods

### *Materials*

Becton Dickinson (Oxnard, CA, USA) supplied 96-well polyvinyl chloride (PVC) microtitre plates (Falcon Micro Test III flexible assay plates). Bovine serum albumin (BSA, fraction V), polyoxyethylene sorbitan monolaurate (Tween 20), goat anti-mouse IgG horseradish peroxidase conjugate (GAM-HRP), goat anti-rabbit IgG-HRP conjugate (GAR-HRP), normal mouse serum (NMS), normal goat serum (NGS), *o*-phenylenediamine dihydrochloride (OPD) and streptavidin-HRP (STV-HRP) were from Sigma Chemical (St. Louis, MO, USA). D-Biotinyl-6-aminocaproic acid *N*-hydroxysuccinimide ester was from Societá Prodotti Antibiotici (Milan, Italy). Enhanced chemiluminescence (ECL) Western blotting kit was from Amersham Italia SRL (Milan, Italy). Milk 'Humana 3' was from Humana Italia (Milan, Italy). CHP-134 neuroblastoma cells were obtained from Dr G Della Valle (University of Pavia, Italy). CHP-134 cell supernatants were harvested from confluent CHP-134 cells, cultured in RPMI, 20% fetal calf serum (FCS), 2 mM glutamine, 100 U $ml^{-1}$ penicillin, 100 $\mu g$ $ml^{-1}$ streptomycin, 25 $\mu g$ $ml^{-1}$ amphotericin B, at 37°C, 5% carbon dioxide.

Phaeochromocytoma and VIPoma heat-stable fractions (HSFs) were prepared as follows: tissues were frozen in liquid nitrogen immediately after surgical excision, lyophilised and homogenised in distilled water. The homogenate was boiled for 6 min and centrifuged at 120 000 *g* for 30 min. The supernatants containing CgA and CgB, which are heat stable (Rosa and Gerdes, 1994), were kept at −20°C until use. Protein concentration was measured using the 'BioRad Protein Assay' kit.

Peripheral blood from healthy controls ($n=22$) was anticoagulated with EDTA (5 mM) or sodium citrate (129 mM), kept in ice and centrifuged at 900 *g* at 4°C for 20 min. Serum samples were also prepared from the blood of the same subjects ($n=22$). Preoperative ($n=7$) and post-operative ($n=2$) sera were obtained from patients with documented phaeochromocytoma. All plasma and serum samples were stored at $-20$°C.

*Anti-chromogranin antibodies*

Monoclonal antibody (MAb) A11 (anti-CgA) and MAb B11 (anti-CgB) were prepared from one mouse immunised with phaeochromocytoma heat-stable fractions (HSFs) (Pelagi *et al.*, 1989). MAb B4E11 was prepared as follows: one BALB/c mouse was immunised by injecting, intraperitonally (i.p.), 50 μg of VIPoma HSF emulsified 1:1 with complete Freund's adjuvant. At 15 day intervals the animal was boosted by injecting 50 μg of VIPoma HSF, i.p. in incomplete Freund's adjuvant (one boost) and in phosphate-buffered saline solution (PBS) (0.15 M sodium chloride, 0.05 M sodium phosphate buffer, pH 7.3) (four boosts). Three days after the last boost, the mouse was sacrificed. Spleen cells were isolated and fused with P3-X63 Ag8-NS1 myeloma cells to generate hybridomas, according to standard procedures (Galfrè and Milstein, 1981).

Hybridomas secreting anti-chromogranin A antibodies were screened by ELISA as described (Pelagi *et al.*, 1989). One clone, named B4E11, was selected for further characterisation and use.

Anti-phaeochromocytoma HSF IgGs were obtained from the sera of two mice immunised with phaeochromocytoma HSF as described (Pelagi *et al.*, 1989). The serum pool (1.1 ml) was diluted 1:5 with 1.5 M glycine, 4 M sodium chloride, pH 9.0 and loaded on a Protein A–Sepharose column pre-equilibrated with the same buffer. After washing, bound IgGs were desorbed with 0.1 M sodium citrate pH 3.0, neutralised and precipitated with 313 mg $ml^{-1}$ ammonium sulphate. The product (327 μg protein) was dialysed against water and stored at $-20$°C.

Monoclonal and polyclonal IgGs were biotinylated as follows: 1 ml and 0.3 ml aliquots, respectively, of 1 mg $ml^{-1}$ antibody solutions in water were mixed with 1 M sodium carbonate buffer, pH 8.8 (0.1 M final concentration), and with a 1 mg $ml^{-1}$ D-biotinyl-6-aminocaproic acid *N*-hydroxysuccinimide ester solution in dimethyl sulphoxide (DMSO) (0.09 mg $ml^{-1}$ final concentration). After incubation for 4 h at room temperature, the solutions were mixed with 1 M lysine (92 mM final concentration) and further incubated 48 h at 4°C. Each product was dialysed overnight against 0.15 M sodium chloride, 0.05 M sodium phosphate, pH 7.3 (PBS) and stored as stock solution at $-20$°C.

*Purification of CgA and CgB*

CgA and CgB were purified from phaeochromocytoma HSF as follows: two columns bearing MAb A11 or B11 were prepared by coupling 3 mg of antibody to 1 g of activated CH–Sepharose (Pharmacia), using 0.1 M sodium carbonate, pH 8.0, as coupling buffer (1 h at 4°C), and 0.1 M Tris–HCl as blocking agent (1 h at 4°C). After column washings (three times with 0.5 M sodium chloride, 0.1 M Tris–HCl buffer, pH 8.0, and with 0.5 M sodium chloride, 0.1 M sodium acetate buffer, pH 8.0) phaeochromocytoma HSF, 1 mg in 3 ml of PBS, was loaded onto the MAb A11–agarose column and washed with PBS until the absorbance of the effluent reached the base line. The column was then eluted with 0.5 M sodium chloride, 0.2 M glycine, pH 2.0. Peak fractions containing CgA were identified by M-ELISA (see below). The protein content in the starting material and in the purified fraction was quantified using the 'BioRad Protein Assay' kit. About 20% of total protein loaded onto the column was recovered in the bound fraction. Moreover, detection of CgA by P-ELISA (see below) showed that more than 95% of immunoreactivity present in the starting material was recovered in the bound fraction and less than 1% in the unbound fraction. After pH neutralisation, the CgA fraction was kept at $-20$°C. CgB was purified from phaeochromocytoma HSF, CgA-depleted fraction, using the B11–agarose column essentially as described for CgA.

*CgA M-ELISA and P-ELISA*

CgA was detected using two analytical systems called M-ELISA and P-ELISA. Schematic representations of CgA M-ELISA and P-ELISA are shown in Figure 1. PVC microtitre plates were coated with B4E11 (10 μg $ml^{-1}$ in PBS, 50 μl per well, overnight at 4°C). All subsequent steps were carried out at room temperature. After washing three times with PBS, the plates were blocked with 3% bovine serum albumin (BSA) in PBS (200 μl per well for 2 h) and washed with PBS again. CgA standard or sample solutions, diluted 1:2 in PBTN (PBS containing 0.5% BSA, 0.05% Tween 20, and 2.5% NGS) were added (50 μl per well) and incubated for 2 h. The plates were washed eight times by emptying and filling with PBS containing 0.05% (v/v) Tween 20 (PBS-T), and incubated with biotinylated MAb A11, 2 μg $ml^{-1}$ (M-ELISA) or biotinylated mouse anti-phaeochromocytoma HSF IgGs, 5 μg $ml^{-1}$ (P-ELISA), both in PBTN (50 μl per well for 1.5 h). The plates were washed again with PBS-T and further incubated with STV–HRP (1:1000 in PBTN, 50 μl per well for 1 h). After the final wash, the plates were incubated with 0.4 mg $ml^{-1}$ *o*-phenylenediamine solution in 0.05 M phosphate–citrate buffer (pH 5.0) containing 3.5 mM hydrogen peroxide (100 μl per well for 45 min). The reaction was stopped by adding 10% (v/v) sulphuric acid (100 μl per well) and the absorbance of each well was read at 492 nm. Each assay was calibrated with eight phaeochromocytoma HSF solutions at various concentrations. The results were

**Figure 1** Calibration curves of CgA M-ELISA and P-ELISA. Insets: schematic representations of the assays.

calculated considering that 20% of total protein in this extract is antigenically related to CgA, as judged from immunoaffinity purification recovery (see above). Detection of CgA antigen in this extract by P-ELISA at various dilutions (1:4000, 1:8000, 1:16 000, 1:32 000), using a calibration curve set up with purified CgA, was 24.5 ± 0.89% of total protein in agreement with previous results. This also indicates a good parallelism of response between crude and purified CgA.

*Western blot analysis*

Sodium dodecylsulphate-polyacrylamide gel electrophoresis (SDS–PAGE) was carried out in a Phast System apparatus (Pharmacia) using ready made polyacrylamide gels (Phast Gels 12.5%, Pharmacia). Samples were two-fold diluted with 20 mM Tris–HCl, pH 8.0, containing 2 mM EDTA, 5% (w/v) SDS, 10% (v/v) β-mercaptoethanol, 0.02% (w/v) bromophenol blue, and boiled for 3 min before electrophoresis. Western blot analysis was carried out essentially as follows: proteins, after SDS-PAGE, were electrophoretically transferred to nitrocellulose membranes using 20 mA for 25 min, and 25 mM Tris, 150 mM glycine, 20% (v/v) methanol (pH 8.9) as transfer buffer. The nitrocellulose membranes were rinsed twice with PBS and were incubated overnight at 4°C in PBS containing 1% BSA and 3% milk. The membranes were then incubated for 2 h with anti-CgA or anti-CgB MAbs (2 $\mu$g ml$^{-1}$) in PBS containing 1% BSA, 3% milk, 1% NGS (PBMN). After washing with PBS containing 0.02% (v/v) Tween 20, the membranes were further incubated for 2 h with GAM–HRP (1:1000) in PBMN. After the final wash, the visualisation reaction was carried out with 'ECL Western Blotting' kit (Amersham), based on luminol substrate and a chemiluminescent principle.

*Size exclusion HPLC*

HPLC gel filtration (SE–HPLC) was carried out at room temperature using a BioSil 250 Guard column joined to a BioSil SEC-250 column (BioRad) as follows: the column was equilibrated and eluted with PBS containing 0.5% BSA (flow rate 0.6 ml min$^{-1}$). Fractions (0.3 ml) were collected and stored at −20°C until analysis. The column was calibrated using thyroglobulin (670 kDa), IgG (158 kDa), bovine serum albumin (66 kDa), ovalbumin (44 kDa), myoglobin (17 kDa) and cyanocobalamin (1.3 kDa), as molecular markers.

*Stability of CgA forms*

To evaluate the stability of CgA forms, aliquots of phaeochromocytoma plasma samples were thawed, incubated at −20°C, 4°C and 37°C for 72 h and further frozen at −20°C. Each aliquot was then thawed, filtered through a 0.45 $\mu$m filter and analysed by SE–HPLC. Each chromatographic fraction was analysed by P-ELISA.

### Results

*CgA sandwich ELISAs*

Monoclonal as well as polyclonal reagents were used to develop sandwich ELISAs for measuring CgA. In particular, two sandwich ELISAs for CgA, called CgA M-ELISA and CgA P-ELISA, were developed. A schematic representation of each assay is depicted in Figure 1. As shown, CgA M-ELISA is based on a sandwich reaction between two monoclonal antibodies (B4E11 and A11), while the CgA P-ELISA is based on one monoclonal antibody (B4E11) and biotinylated mouse anti-phaeochromocytoma HSF polyclonal IgGs.

To verify the specificity of each assay for CgA, the reactivity of purified CgA and CgB was analysed. The cross-reactivity of CgB in the CgA M-ELISA and P-ELISA was less than 0.1%, indicating that these assays can be reliably used for measuring CgA even in the presence of CgB.

*Detection of CgA antigens in serum and plasma*

The CgA content in the serum and plasma of human cancer patients was first measured by P- and M-ELISA. In agreement with previous findings (O'Connor and Bernstein, 1984; O'Connor and Deftos, 1986), the results indicate that CgA levels, as measured by both assays, are increased in phaeochromocytoma patients in comparison with normal subjects (Table I). Moreover, CgA levels decreased to normal values after tumour surgery, suggesting that CgA antigen, as measured by P- and M-ELISA, could be a useful tumour marker. However, the ratio between CgA values measured by M- and P-ELISA ('P/M ratio') was different in different patients ranging from 0.45 to 2.8. This suggests that the biological fluids examined contain different antigenic forms that are differentially recognised by different assays. Of note, some variability in the P/M ratio was observed, though to a lower extent, within serum samples from normal subjects (from 1.5 to 2.12) and plasma samples (from 1.3 to 2.1).

Assay validation studies showed that P-ELISA intra-assay ($n=10$) and interassay ($n=6$) coefficient of variation (CV), was 5.2% and 22.5%, respectively, as determined using ten serum samples from normal subjects and patients. Similarly, M-ELISA intra-assay CV was 6.3%, while interassay CV was 19.5%. To evaluate the parallelism of response between sera and standards, four serum samples were analysed at 1:3 and 1:6 dilution. The values obtained by P-ELISA and M-ELISA at 1:6 dilution were 96.0 ± 3.9% and 121 ± 47.8%

**Table I** Detection of CgA in the serum and plasma of normal subjects and of phaeochromocytoma patients

| *Sample* | *CgA (ng ml$^{-1}$, mean ± s.d.)* *M-ELISA* | *P-ELISA* | *P/M* |
|---|---|---|---|
| *Normal subjects (n=22)* | | | |
| (S) (serum) | 24 ± 6.4 | 43.2 ± 6.4 | 1.79 ± 0.2 |
| (P) (plasma-citrate) | 25 ± 8.6 | 43.4 ± 16 | 1.72 ± 0.2 |
| (P) (plasma-EDTA) | 33 ± 9.6 | 50.2 ± 28.2 | 1.49 ± 0.45 |
| *Phaeochromocytoma patients (n=9)* | | | |
| S1 | 80.4 | 168.8 | 2.1 |
| S2 | 48.2 | 120 | 2.5 |
| S3 | 90.2 | 45 | 0.5 |
| S3p | 17.6 | 8 | 0.45 |
| S4 | 1866 | 3108 | 1.66 |
| S5 | 32.2 | 29.8 | 0.92 |
| S6 | 20.4 | 52.8 | 2.58 |
| S7 | 19.8 | 50 | 2.5 |
| S7p | 5 | 14 | 2.8 |
| P1 | 220 | 560 | 2.54 |
| P2 | 110 | 260 | 2.36 |

p, After surgery.

(mean ± s.d.) respectively, of the values obtained at 1:3 dilution. Thus, while a good parallelism of response was observed with P-ELISA, the effect of dilution on the analytical recovery obtained by M-ELISA was more variable and dependent on serum samples.

This also suggests that different samples contain forms with different immunoreactivity with MAb A11 and B4E11.

*Characterisation of circulating CgA forms*

To characterise the molecular forms of circulating CgA and to investigate the cause for the discrepancies observed with different assays, the molecular weight of serum and tissue CgA was characterised by SE-HPLC.

As shown in Figure 2, the chromatographic behaviour of CgA present in the serum of different patients was markedly different. In particular, at least three peaks eluting at 600 kDa (CgA-I), 100 kDa (CgA-II) and 55 kDa (CgA-III) were observed, in different proportions, within serum samples from different patients.

Interestingly, the 'P/M ratio' of these sera before chromatography was different, increasing from 0.5 to 2.5 in patients with an increasing proportion of CgA-I. Accordingly, the 'P/M ratio' of isolated fractions ranged from about

**Figure 2** SE-HPLC of three phaeochromocytoma serum samples (S1, S2 and S3, 100 $\mu$l aliquots), phaeochromocytoma HSF (100 $\mu$l, 20 $\mu$g ml$^{-1}$), purified CgA (10 $\mu$g ml$^{-1}$, 100 $\mu$l) and CHP-134 cell supernatant (100 $\mu$l). Fractions were analysed by M-ELISA.

2.5 (CgA-I) to 0.5 (CgA-III), suggesting that CgA-I is detected more efficiently by P-ELISA, whereas CgA-III is detected more efficiently by M-ELISA, when compared with the phaeochromocytoma HFS standard. In conclusion, these results suggest that the discrepancies observed with different assays are related to the presence of variable proportions of three forms of CgA characterised by a different behaviour in SE-HPLC.

Other biological samples with a higher content of CgA were then analysed. Interestingly, CgA forms similar to those found with serum samples were obtained by SE–HPLC of crude phaeochromocytoma HSF (used as reference standards in M- and P-ELISA) and immunopurified CgA, while fresh CHP-134 neuroblastoma cell supernatants contained mainly the CgA-I form (Figure 2, right panels). Also in these cases, the proportion of each form over the total was variable. Moreover, as described above for serum samples, P-ELISA was more efficient in detecting CgA-I, whereas M-ELISA detected more efficiently CgA-III fractions (Figure 3).

Peak fractions were then analysed by SDS–PAGE under non-reducing conditions and Western blotting with MAb B4E11. As shown in Figure 4, the CgA-I and CgA-II obtained by SE–HPLC of phaeochromocytoma HSF and of one serum sample containing 2–3 $\mu$g ml$^{-1}$ CgA (sample S4) were resolved in two main bands of 60–70 kDa (lanes b,d and g). Similarly, the CgA-III was resolved in a main band of about 60 kDa (lanes c and f). Thus, the distinct forms observed by SE–HPLC appear to be made up by polypeptides with a very similar molecular weight. Of note, some bands of lower relative mass probably corresponding to proteolytic fragments, were observed with CgA-I and CgA-II from phaeochromocytoma HSF (lanes b and d) but not with serum or CHP-134 CgA-I (lanes g and h). We do not know the reason for this behaviour. However, one band of approximately 30 kDa was observed after gel-filtration of phaeochromocytoma HSF (lanes b and d), while it was almost absent in the starting material (lane a) suggesting that this band is related to proteolysis occurring after SE–HPLC.

To assess further the immunochemical properties of CgA forms, the relative binding of CgA-I, -II and -III to MAb A11 and B4E11 was also examined. These antibodies recognise epitopes located within residues 68–70 (GAK) and 81–90 (GFEDELSEVL) of CgA, respectively, as mapped using 20 peptides covering most of the CgA sequence and 12 overlapping peptides encompassing the CgA(65–96) sequence (A Corti et al., 1996). Thus, these antibodies could be used to probe the cognate epitopes in CgA forms. For this purpose, SE–HPLC fractions

**Figure 3** SE-HPLC of a phaeochromocytoma HSF solution (100 $\mu$l, 20 $\mu$g ml$^{-1}$) (**a** and **c**) and a serum sample (S4, 100 $\mu$l) of a phaeochromocytoma patient (**b** and **d**). Fractions were analysed by CgA M-ELISA (**a** and **b**), CgA P-ELISA (**c** and **d**).

corresponding to CgA-I, -II and -III were incubated with A11- and B4E11-coated plates. The total bound antigenic material was detected with a rabbit polyclonal anti-CgA antibody and quantified using a calibration curve made up with phaeochromocytoma HSF solutions. As shown in Table I, the relative immunoreactivity of these fractions to A11 and B4E11 was different, suggesting that, in spite of all CgA forms expressing both epitopes, the accessibility or the conformation of the cognate epitopes in these forms are different.

In conclusion, CgA-I, CgA-II and CgA-III, although appearing to be made up of polypeptides with similar molecular weight, are characterised by markedly different hydrodynamic properties and different immunoreactivity.

To investigate whether CgA-I is related to multimeric complexes, other experiments were undertaken. The results can be summarised as follows: a series of ELISA experiments carried out using solid-phase B4E11 in the capturing step and biotinylated B4E11 in the detection step (homosandwich ELISA) failed to detect polyvalent aggregated material both

**Figure 4** Analysis of SE-HPLC fractions by Western blotting with MAb B4E11. Two aliquots (100 $\mu$l) of phaeochromocytoma HSF solutions (20 $\mu$g ml$^{-1}$ and 1.18 mg ml$^{-1}$ in PBS) were gel filtered through a Bio-Sil SEC-250 column as described in 'Materials and methods' and analysed by M-ELISA. Peak fractions were then subjected to SDS–PAGE and Western blotting with MAb B4E11. Lane a, phaeochromocytoma HSF (starting material (s.m.) of SE-HPLC); lanes b and c, gel filtration of 20 $\mu$g ml$^{-1}$ phaeochromocytoma HSF; lanes d, e and f, gel filtration of 1.18 mg ml$^{-1}$ phaeochromocytoma HSF. Lanes b and d, CgA-I; lane e, CgA-II; lanes c and f, CgA-III. This figure also shows the Western blotting of CgA-I of serum S4 chromatography (see Figure 7) (lane g); CHP-134 cell supernatant (lane h); and CgA purified by affinity chromatography on A11-agarose from phaeochromocytoma HSF (lane i), both used as starting material for SE–HPLC of Figure 2.

**Figure 5** Sandwich ELISA of phaeochromocytoma HSF (**a** and **b**), phaeochromocytoma HSF treated with DSS (**c**), and serum S4 (**d** and **e**) using B4E11 or A11 as capturing antibodies and biotin-B4E11 or biotin-A11 as detecting reagents, in both combinations. The sandwich ELISAs were carried out using the same conditions (buffers and incubation times) as described for M-ELISA (see Materials and methods) except that for the experiments reported in b and e Tween 20 was omitted from all incubation and washing buffers. Phaeochromocytoma HSF was treated with DSS as follows: 25 μl of phaeochromocytoma HSF (40 $\mu$g ml$^{-1}$) was mixed with 1.25 $\mu$l of 25 mM DSS and incubated for 30 min at 25°C. Then 3 $\mu$l of 1 M ammonium acetate was added to block the cross-linking reaction.

in the S4 serum sample and in a phaeochromocytoma HSF solution (Figure 5a and d), even when detergents were omitted in the assay incubation and washing buffers (Figure 5b and e). The same results were obtained using MAb A11 in a similar homosandwich ELISA, whereas strong signals were obtained with control heterosandwich ELISA made up with solid-phase MAb B4E11 and biotinylated A11 as well as with solid phase A11 and biotinylated B4E11 (Figure 5a, b, d and e). To rule out the possibility that complex dissociation occurred during assay incubation, phaeochromocytoma HSF was treated with disuccinimidyl suberate (DSS), a bifunctional reagent widely used to cross-link protein complexes, and analysed again by the homo- and hetero-sandwich ELISAs. Also in this case (Figure 5c) no signals were detected by the homosandwich ELISAs. Moreover, no bands corresponding to high molecular weight complexes or aggregates of CgA were observed by Western blotting analysis of phaeochromocytoma HSF treated with DSS.

Thus, serum samples as well as tissue extracts contain at least three forms of CgA that appear to be monomeric proteins.

*Stability of circulating CgA forms*

The stability of circulating CgA forms was then investigated. To this purpose, two phaeochromocytoma plasma samples, containing mainly CgA-II and a small amount of CgA-I, were incubated at various temperatures (−20°C, 4°C and

Figure 6 Gel filtration chromatography of two phaeochromocytoma plasma samples (P1 and P2) after 72 h incubation at various temperatures. Fractions were analysed by CgA P-ELISA.

**Table II** CgA antigen in phaeochromocytoma SE-HPLC fractions as measured by ELISA using plates coated with B4E11 and A11[a]

| *SE-HPLC fraction*[b] | *CgA* ($\mu$g ml$^{-1}$) *A11* | *B4E11* | *B4E11/A11* |
|---|---|---|---|
| CgA-I | 28.0 ± 0.7 | 12.2 ± 0.5 | 0.43 |
| CgA-II | 8.95 ± 0.85 | 6.55 ± 0.15 | 0.73 |
| CgA-III | 4.2 ± 0.2 | 5.9 ± 0.9 | 1.40 |

[a] The ELISA was carried out essentially as described for P-ELISA, except that plates were coated with A11 or B4E11 and bound CgA was detected with a rabbit polyclonal anti-phaeochromocytoma serum (1:5000) followed by a goat anti-rabbit HRP conjugate. [b] CgA fractions were obtained by SE-HPLC of phaeochromocytoma HSF.

37°C) for 72 h and subsequently analysed by SE-HPLC and P-ELISA. As shown in Figure 6 , a small increase of CgA-I was observed after incubation at 37°C while a decrease was observed at 4°C. This indicates that CgA forms may change as a function of temperature and that CgA-I is unlikely to be a proteolytic precursor of CgA-II.

### Discussion

In this work we have characterised the structure of circulating CgA of phaeochromocytoma patients and compared it with that of CgA extracted from tumour tissues. The study was carried out using: (1) various ELISAs set up with monoclonal and polyclonal antibodies; (2) SE-HPLC, coupled to ELISA detection of fractions; and (3) Western blotting analysis of chromatographic fractions with a high affinity anti-CgA monoclonal antibody (B4E11).

In accordance with previous findings (O'Connor and Deftos, 1987), circulating CgA was found to be heterogeneous. In particular, CgA was found to circulate in at least three different antigeneic forms of 600 kDa, 100 kDa and 55 kDa, by SE-HPLC (here called CgA-I, CgA-II and CgA-III, respectively). The proportion of each form in various patients was variable. For instance, we found that the serum of some patients contained only the 600 kDa or the 55 kDa form, whereas that of other patients contained mixtures of all forms. These forms were differentially detected by two immunoassays based on monoclonal and polyclonal antibodies. Moreover, although all these forms express the epitopes recognised by two monoclonal antibodies (A11 and B4E11), their relative immunoreactivity with these MAbs was different. Western blotting experiments showed that CgA-I, CgA-II and CgA-III are resolved in one or two main bands corresponding to polypeptides of similar molecular weight (60-70 kDa). Other minor bands likely corresponding to proteolytic fragments can also be observed. Although proteolytic processing could contribute to the observed hydrodynamic behaviour of CgA forms, these results suggest that the markedly distinct hydrodynamic and immunological properties of CgA forms are not simply related to proteolytic fragmentation.

The possibility exists that CgA-I and CgA-II correspond to multimeric complexes or aggregates. However, we think that this hypothesis is unlikely for various reasons:

(1) It has been shown previously that CgA aggregates, thought to be present within the secretory granules, are characterised by an apparent molecular weight, by gel filtration, much higher than that observed for CgA-I and CgA-II (> 1500 kDa) (Yoo and Albanesi, 1990).
(2) The same report showed that, after aggregate dissociation, CgA still behaves as a large protein close to the CgA-I of this study.
(3) We were unable to detect multimeric complexes in serum and tissue samples by several analyses based on immunological and biochemical methods (see Results).
(4) No CgA binding proteins have been detected in serum by previous studies (Takiyyuddin *et al.*, 1990).

Although the presence of multimeric complexes in serum cannot be completely ruled out, we think that other factors must be taken into account to explain the behaviour of circulating CgA in gel filtration.

Previous studies have shown that CgA isolated from chromaffin granules elutes from a column of Sephadex G-200 with an apparent molecular weight close to that of CgA-I, whereas the same material in the same buffer, shows an apparent molecular weight of 77 kDa by sedimentation analysis (Smith and Winkler, 1967). Similar results have also been reported by other researchers with purified CgA (Fisher-Colbrie and Schober, 1987). This unusual behaviour of CgA in gel filtration has been associated with the conformation of this protein approaching that of a random coil polypeptide and behaving like a large expanded sphere with an effective hydrodynamic radius of 77 Å.

Thus, the occurrence of a CgA form in serum with an apparent molecular weight of 600 kDa may be simply explained by the fact that we used globular proteins to calibrate the column with presumably smaller hydrodynamic sizes. However, this would imply that serum also contains other forms with different conformations and hydrodynamic sizes like those found in CgA-II and CgA-III, still made up of polypeptides of 60-70 kDa.

The different relative immunoreactivity of these forms observed with B4E11 and A11, directed against two adjacent epitopes located within residues 68-70 and 81-90, respectively, could also reflect different epitope conformations in these proteins.

The finding that serum contains CgA forms with apparently different molecular sizes may reflect the existence of similar forms also in tissues. Accordingly, CgA forms with large (80 Å) and small (54 Å) Stokes radius have been identified in the adrenal medulla and in the brain, respectively (O'Connor and Frigon, 1984).

Evidence for differential proteolytic processing of CgA in different endocrine cells has been reported (Deftos *et al.*, 1990, Brandt *et al.*, 1994). Interestingly, in these reports, gel filtration chromatography studies showed multiple and distinct immunoreactive species of CgA in different cell lines. Although the comparison with our serum CgA forms is difficult, since the molecular weights were not estimated, it is interesting to note that also in this case forms with apparently larger than the $^{125}$I-CgA standard, used to calibrate the column, were observed in some cell lines. In view of the hypothesis that CgA is a multihormone precursor, the occurrence of forms with different hydrodynamic properties could have important implications in the regulation of the proteolytic processing of these proteins in different tissues or in blood. For instance, it is possible that specific proteolytic sites are masked or exposed in different forms.

Work aimed at investigating the sensitivity of CgA forms to proteases and at elucidating the factors that affect their hydrodynamic behaviours could help in a better understanding of the complex structure-function relationships of chromogranins and of their physiological and pathological roles in normal subjects and tumour patients.

In conclusion, these results suggest that circulating CgA in phaeochromocytoma patients consists of at least three antigenic forms that appear to be made up of polypeptides with similar molecular weight and different hydrodynamic properties and immunoreactivity. Since the ratio of CgA concentrations measured by P- and M-ELISA (P/M ratio) is dependent on the proportion of these forms over the total, differential detection of CgA with these assays in a high number of patients with different tumours may be exploited to study the diagnostic potential of these forms.

**Abbreviations**

CgA, chromogranin A; CgB, chromogranin B; MAb, monoclonal antibody; HSF, heat-stable fraction; SE-HPLC, size exclusion high-performance liquid chromotography

**Acknowledgements**
The authors wish to thank Barbara Colombo and Angelina Sacchi for excellent technical assistance, and Dr Roberto Buffa for kindly providing phaeochromocytoma serum samples. Moreover, we thank the 'Ministero dell'Universitá e della Ricerca Scientifica e Tecnologica' of Italy for supplying MAb A11 and B11. The work was supported by a grant from the 'Associazione Italiana per la Ricerca sul Cancro'.

### References

BENEDUM UM, LAMOUROUX A, KONECKI DS, ROSA P, HILLE A, BAEUERLE PA, FRANK R, LOTTSPEICH F, MALLET J AND HUTTNER WB. (1987). The primary structure of human secretogranin I (chromogranin B); comparison with chromogranin A reveals homologous terminal domains and a large intervening variable region. *EMBO J.*, **6**, 1203–1211.

BRANDT DW, BURTON DW, HOGUE-ANGELETTI R AND DEFTOS LJ. (1994). Chromogranin A peptide-specific antisera and high-performance size exclusion chromatography demonstrate amino-terminal and carboxy-terminal fragments of the native molecule in human cell lines. *Proc. Soc. Exp. Biol. Med.*, **205**, 316–320.

CORTI A, LONGHI R, GASPARRI A, CHEN F, PELAGI M AND SICCARDI AG. (1996). Antigenic regions of human chromogranin A and their topographic relationships with structural/functional domains. *Eur. J. Biolchem.*, **235**, 275–280.

DEFTOS LJ. (1991). Chromogranin A: its role in endocrine function and as an endocrine and neuroendocrine tumour marker. *Endocrinol. Rev.*, **12**, 181–187.

DEFTOS LJ, LINNOILA RI, CARNEY DN, BURTON DW, LEONG SS, O'CONNOR DT, MURRAY SS AND GAZDAR AF. (1988). Demonstration of chromogranin A in human neuroendocrine cell lines by immunohistology and immunoassay. *Cancer*, **62**, 92–97.

DEFTOS LJ, GAZDAR AF, HOGUE-ANGELETTI R, MULLEN PS AND BURTON DW. (1990). Distinct patterns of chromogranin A-related species can be demonstrated in endocrine cells. *Bone Mineral.*, **9**, 169–178.

FISHER-COLBRIE R AND SCHOBER M. (1987). Isolation and characterization of chromogranins A, B and C from bovine chromaffin granules and a rat pheochromocytoma. *J. Neurochem.*, **48**, 262–270.

GALFRÈ G AND MILSTEIN C. (1981). Preparation of monoclonal antibodies: strategies and procedures. *Methods Enzymol.*, **73**, 3–46.

HELLE KB AND ANGELETTI RH. (1994). Chromogranin A: a multipurpose prohormone? *Acta Physiol. Scand.*, **152**, 1–10.

HELMEN LJ, AHN TG, LEVINE MA, ALLISON A, COHEN PS, COOPER MJ, COHN DV AND ISRAEL MA. (1988). Molecular cloning and primary structure of human chromogranin A (secretory protein I) cDNA. *J. Biol. Chem.*, **263**, 11559–11563.

HSIAO RJ, SEEGER RC, YU AL AND O'CONNOR DT. (1990). Chromogranin A in children with neuroblastoma: serum concentration parallels disease stage and predicts survival. *J. Clin. Invest.*, **85**, 1555–1559.

JOHNSON PWM, JOEL SP, LOVE S, BUTCHER M, PANDIAN MR, SQUIRES L, WRIGLEY PFM AND SLEVIN ML. (1993). Tumour markers for prediction of survival and monitoring of remission in small cell lung cancer. *Br. J. Cancer.*, **67**, 760–766.

KONECKI DS, BENEDUM UM, GERDES HH AND HUTTNER WB. (1987). The primary structure of human chromogranin A and pancreastatin. *J. Biol. Chem.*, **262**, 17026–17030.

METZ-BOUTIGUE MH, GARCIA-SABLONE P, HOGUE-ANGELETTI R AND AUNIS D. (1993). Intracellular and extracellular processing of chromogranin A. *Eur. J. Biochem.*, **217**, 247–257.

O'CONNOR DT AND BERNSTEIN KN. (1984). Radio immunoassay of chromogranin A in plasma as a measure of exocytotic sympathoadrenal activity in normal subjects and patients with pheochromocytoma. *N. Engl. J. Med.*, **311**, 764–770.

O'CONNOR DT AND DEFTOS LJ. (1986). Secretion of chromogranin A by peptide-producing endocrine neoplasms. *N. Eng. J. Med.*, **314**, 1145–1151.

O'CONNOR DT AND DEFTOS LJ. (1987). How sensitive and specific is measurement of plasma chromogranin A for the diagnosis of neuroendocrine neoplasia? *Ann. NY. Acad. Sci.*, **493**, 379–386.

O'CONNOR DT AND FRIGON RP. (1984). Chromogranin A, the major catecholamine storage vesicle soluble protein. *J. Biol. Chem.*, **259**, 3237–3247.

PELAGI M, BISIANI C, GINI A, BONARDI MA, ROSA P, MARÉ P, VIALE G, COZZI MG, SALVADORE M, ZANINI A, SICCARDI AG AND BUFFA R. (1989). Preparation and characterization of anti-human chromogranin A and B (secretogranin I) monoclonal antibodies. *Mol. Cell. Probes*, **3**, 87–101.

ROSA P AND GERDES HH. (1994). The granin protein family: markers for neuroendocrine cells and tools for the diagnosis of neuroendocrine tumors. *J. Endocrinol. Invest.*, **17**, 207–225.

SIMON JP AND AUNIS D. (1989). Biochemistry of the chromogranin A protein family. *Biochem. J.*, **262**, 1–13.

SMITH AD AND WINKLER H. (1967). Purification and properties of an acidic protein from chromaffin granules of bovine adrenal medulla. *Biochem. J.*, **103**, 483–492.

SOBOL RE, O'CONNOR DT, ADDISON J, SUCHOCKI K, ROYSTON I AND DEFTOS LJ. (1986). Elevated serum chromogranin A concentrations in small-cell lung carcinoma. *Ann. Intern. Med.*, **105**, 698–700.

TOTSCH M, MULLER LC, HITTMAIR A, OFNER D, GIBBS AR AND SCHMID KW. (1992). Immunohistochemical demonstration of chromogranins A and B in neuroendocrine tumours of the lung. *Hum. Pathol.*, **23**, 312–316.

TAKIYYUDDIN MA, CERVENKA JH, HSIAO RJ, BARBOSA JA, PARMER RJ AND O'CONNOR T. (1990). Chromogranin A. Storage and release in hypertension. *Hypertension*, **15**, 234–246.

WEILER R, FEICHTINGER H, SCHMID KW, FISCHER-COLBRIE R, GRIMELIUS L, CEDERMARK B, PAPOTTI M, BUSSOLATI G AND WINKLER H. (1987). Chromogranin A and B and secretogranin II and in bronchial and intestinal carcinoids. *Virchows. Arch. A.*, **412**, 103–109.

WINKLER H AND FISCHER-COLBRIE R. (1992). The chromogranins A and B: the first 25 years and future perspectives. *Neuroscience*, **49**, 497–528.

WU HJ, ROZANSKY DJ, PARMER RJ, GILL BM AND O'CONNOR DT. (1991). Structure and function of the chromogranin A gene. *J. Biol. Chem.*, **266**, 13130–13134.

YOO SH AND ALBANESI JP. (1990). $Ca^{2+}$-induced conformational change and aggregation of chromogranin A. *J. Biol. Chem.*, **265**, 14414–14421.